# Novel VCP modulators mitigate major pathologies of rd10, a mouse model of retinitis pigmentosa

**DOI:** 10.1038/srep05970

**Published:** 2014-08-06

**Authors:** Hanako Ohashi Ikeda, Norio Sasaoka, Masaaki Koike, Noriko Nakano, Yuki Muraoka, Yoshinobu Toda, Tomohiro Fuchigami, Toshiyuki Shudo, Ayana Iwata, Seiji Hori, Nagahisa Yoshimura, Akira Kakizuka

**Affiliations:** 1 Department of Ophthalmology and Visual Sciences, Kyoto University Graduate School of Medicine, Kyoto 606-8501, Japan; 2 Laboratory of Functional Biology, Kyoto University Graduate School of Biostudies, Kyoto 606-8501, Japan; 3 Center for Anatomical Studies, Kyoto University Graduate School of Medicine, Kyoto 606-8501, Japan; 4 Daito Chemix, Ishibashi-cho Fukui-city Fukui 910-3137, Japan

## Abstract

Neuroprotection may prevent or forestall the progression of incurable eye diseases, such as retinitis pigmentosa, one of the major causes of adult blindness. Decreased cellular ATP levels may contribute to the pathology of this eye disease and other neurodegenerative diseases. Here we describe small compounds (Kyoto University Substances, KUSs) that were developed to inhibit the ATPase activity of VCP (valosin-containing protein), the most abundant soluble ATPase in the cell. Surprisingly, KUSs did not significantly impair reported cellular functions of VCP but nonetheless suppressed the VCP-dependent decrease of cellular ATP levels. Moreover, KUSs, as well as exogenous ATP or ATP-producing compounds, e.g. methylpyruvate, suppressed endoplasmic reticulum stress, and demonstrably protected various types of cultured cells from death, including several types of retinal neuronal cells. We then examined their *in vivo* efficacies in rd10, a mouse model of retinitis pigmentosa. KUSs prevented photoreceptor cell death and preserved visual function. These results reveal an unexpected, crucial role of ATP consumption by VCP in determining cell fate in this pathological context, and point to a promising new neuroprotective strategy for currently incurable retinitis pigmentosa.

Despite recent advances in the development of new drugs, there remain many incurable disorders, e.g. neurodegenerative diseases, ischemic diseases, and chronic inflammation, in which the major pathology in the affected organs is early cell death, which occurs long before the death of the individual. Indeed, currently no drug is available to prevent such early cell death *in vivo*. If such drugs were available, many of these disorders, if not all, might be prevented or delayed. In the 1990s, due to a growing, but still imperfect, understanding of the molecular bases of apoptotic cell death, caspase inhibitors were developed and were heralded as miracle drugs, but they were ineffective in preventing cell death *in vivo*. This might be explained as follows: caspases determine how cells die but are unable to affect the commitment to cell death. Thus, drugs that could delay or prevent the commitment to cell death have been actively pursued.

Retinitis pigmentosa, which is caused by a gradual degeneration and loss of photoreceptors, is another intractable eye disease, and more than 1.5 million patients suffer from progressive visual deterioration with this disorder. Clinical trials using a neurotrophic factor have been initiated^1^. In retinitis pigmentosa, involvement of endoplasmic reticulum (ER) stress has been proposed^2^,^3^. Thus, new drugs or compounds with ER stress-reducing activities may prove to be neuroprotective, and are thus worth investigating for the treatment or prevention of retinitis pigmentosa.

We have long been working to elucidate the molecular bases of polyglutamine diseases, as informative models for neurodegeneration. We and other research groups produced several lines of evidence that implicate valosin-containing protein (VCP), an AAA (ATPases Associated with diverse cellular Activities)-type ATPase with ubiquitous expression, as a major player causing neurodegeneration. It is notable that VCP is highly conserved among species; its amino acid sequences are completely identical among mouse, rat, and human, and 84% identical between human and *Drosophila*^4^. We first assumed that specific cellular genes are involved in the pathogenesis of polyglutamine diseases, and thus had established *Drosophila* models of polyglutamine diseases for genetic analyses. Mutant screening revealed that *Ter94* loss-of-function alleles mitigated polyglutamine-induced eye degeneration^4^. Conversely, overexpression of wild-type *Ter94* enhanced the polyglutamine-induced eye degeneration^4^. Since the mammalian *Ter94* ortholog is *VCP*, these results raised the possibility that VCP is involved in the pathogenesis of human neurodegenerative diseases^5^. Consistent with this possibility, VCP mutations were identified that are causative for IBMPFD (inclusion body myopathy associated with Paget disease of bone and frontotemporal dementia)^6^, a human hereditary disease with dementia, or for rare cases of familial amyotrophic lateral sclerosis (ALS)^7^. In our evaluation, all tested pathogenic VCPs possessed elevated ATPase activities, as compared with wild-type VCP^8^, indicating that the constitutive elevation of its ATPase activity may be pathogenic.

These lines of evidence suggested that inhibitors of the ATPase activity of VCP could protect neuronal cells. In addition to its ATPase activity, however, many cellular functions of VCP have been proposed^9^,^10^,^11^, e.g., proteasome-mediated protein degradation, endoplasmic reticulum-associated degradation (ERAD), cell cycle control, membrane fusion, maintenance of the Golgi apparatus, protein trafficking, autophagy, genomic DNA surveillance, etc., some of which are crucial for cell survival. Indeed, VCP knockdown and overexpression of dominant-negative forms of VCP showed toxicity in cultured cells^12^,^13^. DBeQ, a recently reported VCP inhibitor (*in vitro* IC_50_ (half maximal inhibitory concentration) 1 μM)^14^, also showed cellular toxicity. Given that VCP has multiple cellular functions, some of them would require ATP hydrolysis and others would not. Thus, it is a challenge to find small compounds that can inhibit or reduce the ATPase activity of VCP without incurring the general toxicity caused by loss of crucial cellular functions of VCP.

## Results

### KUSs inhibited VCP ATPase activity but not VCP cellular functions

**Figure 1:**
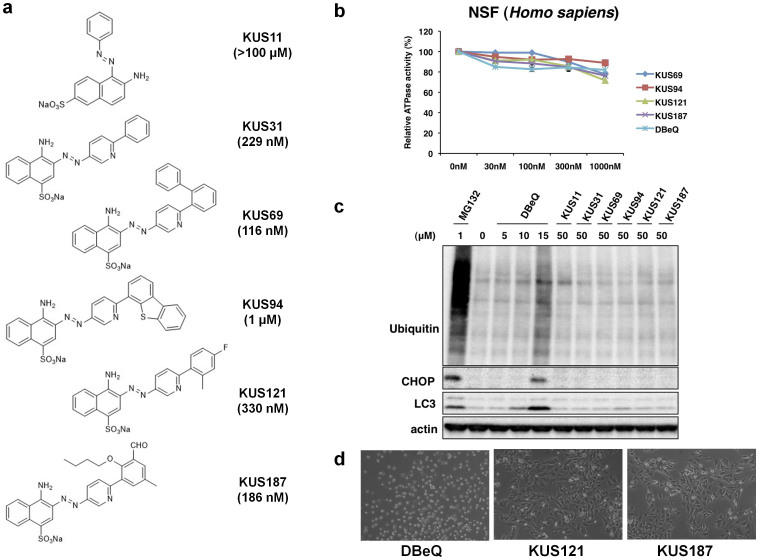
Structures and characterization of KUSs, novel VCP modulators. (a) Structures and IC_50_ values of KUS11, KUS31, KUS69, KUS94, KUS121, and KUS187. Note that KUS11 did not inhibit the ATPase activity of recombinant VCP, and it did not share a common structure with the other KUSs. (b) ATPase activity assays of recombinant human NSF, comparing KUSs and DBeQ. (c) Immunoblot analysis of ubiquitinated proteins, an ER stress marker (CHOP), and an autophagy indicator (LC3), comparing KUSs and DBeQ. As a control, MG132, a proteasome inhibitor, was used for the analysis. Actin served as a loading control. Complete scans of the different blots are presented in Supplementary Fig. 7. (d) Comparison of KUSs and DBeQ for cell death-inducing activities. HeLa cells were treated with 50 μM DBeQ or KUSs for 24 hours.

In our search for novel VCP ATPase inhibitors, we found that a naphthalene derivative can inhibit the ATPase activity of VCP with no apparent toxicity at 10 μM on cultured cells. Based on the chemical structure, about two hundred compounds were newly synthesized and named KUSs (Kyoto University substances). Some of them, e.g. KUS31, 69, 94, 121, and 187, clearly inhibited the ATPase activity of recombinant VCP *in vitro* with IC_50_ values ranging from approximately 100 nM to 1 μM (Fig. 1a). These IC_50_ values were equivalent to or much lower than that of DBeQ^14^, whose reported IC_50_ is 1 μM (Supplementary Fig. 1). It is notable that KUSs very mildly inhibited the ATPase activity of N-ethylmaleimide-sensitive fusion protein (NSF) (Fig. 1b), whose structure is most closely related with that of VCP.

We then examined the effect of KUS31, 69, 94, 121, and 187 on VCP functions in cultured cells. We also used DBeQ as a reference compound. As reported, DBeQ induced accumulation of ubiquitinated proteins, ER stress, autophagy, and eventually cell death (Fig. 1c and d). Surprisingly, these compounds did not induce any of these phenotypes (Fig. 1c and d). These results clearly indicated that ATPase inhibition by KUS31, 69, 94, 121, and 187 (referred to as “KUSs” hereafter, for simplicity) did not interfere with reported cellular VCP functions (referred to as “VCP functions” hereafter), indicating that VCP functions do not necessarily require its ATPase activity (see Discussion).

### KUSs protected cells under ER stress-inducing conditions

**Figure 2:**
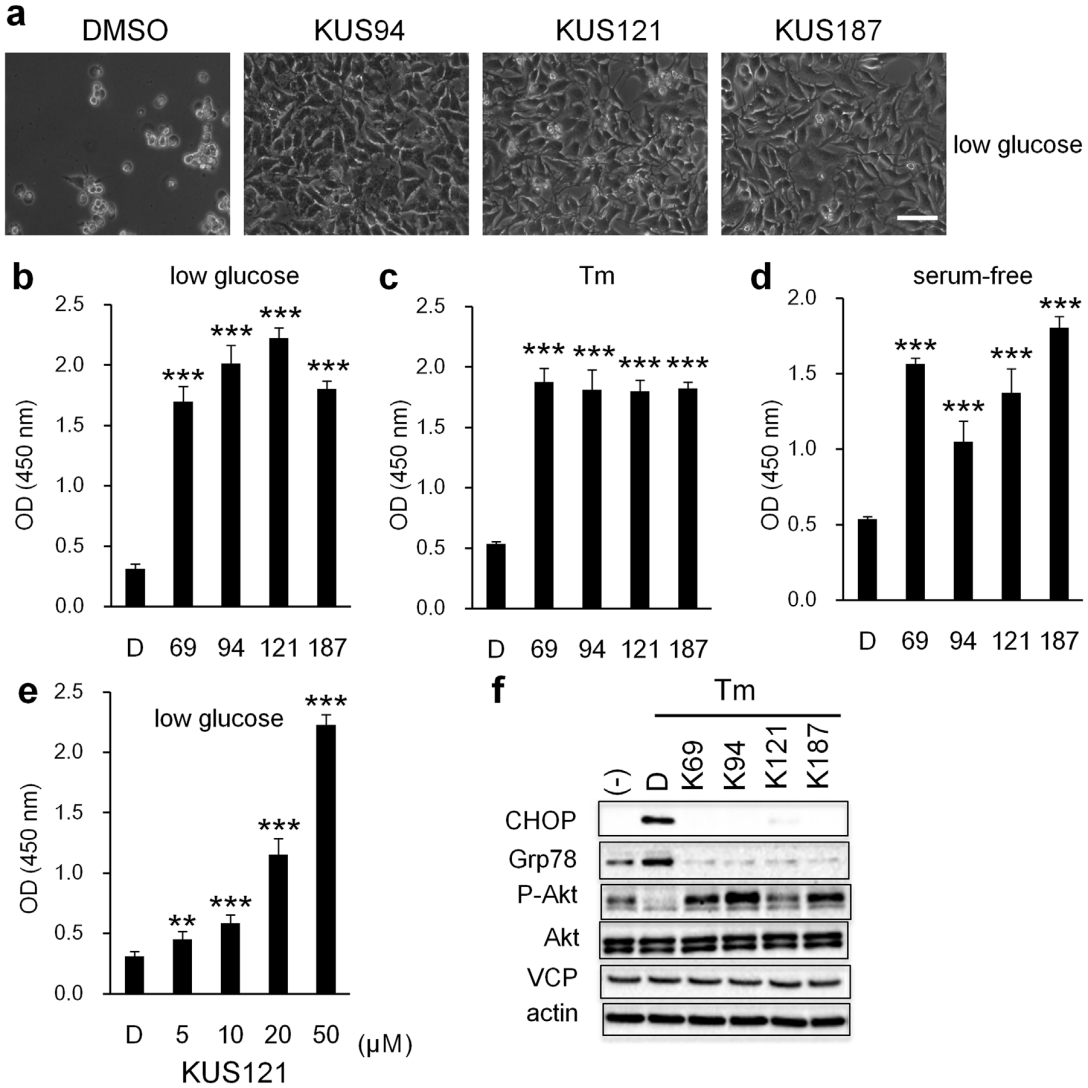
Prevention of cell death and ER stress by KUSs. (a) Photographs of HeLa cells, cultured with DMSO (DMSO) or KUSs (KUS94, KUS121, and KUS187, 20 μM each) for 41 hours in low glucose (0.2 g/l) medium. Scale bar, 100 μm. (b–e) WST (water-soluble tetrazolium salts) values reflecting relative live cell numbers are shown, as optical density (OD) at 450 nm. Error bars indicate SD. (b) WST values of HeLa cells, cultured in low glucose (0.2 g/l) with DMSO (control) or KUSs (50 μM for KUS121; 20 μM for KUS69, KUS94, and KUS187, n = 3) for 41 hours. (c) Cell viability, indicated by WST values of HeLa cells, cultured with tunicamycin (Tm) (0.2 μg/ml) for 41 hours with KUSs (20 μM each, n = 3). (d) WST values of HEK293 cells, cultured under serum-free conditions for 65 hours with DMSO (control) or KUSs (20 μM each, n = 3). *** *P* < 0.001, vs. DMSO control (Dunnett's test). (e) WST values of HeLa cells, cultured in low glucose (0.2 g/l) medium with different concentrations of KUS121 (5, 10, 20, and 50 μM, n = 3) for 41 hours. ** *P* = 0.008, *** *P* < 0.001, vs. DMSO control (Dunnett's test). (f) Western blot analysis of HeLa cells, treated with tunicamycin (Tm, 0.5 μg/ml) with DMSO (control) or KUSs (50 μM each, KUS69, KUS94, KUS121, and KUS187) for 5 hours. Complete scans of the different blots are presented in Supplementary Fig. 8.

Additionally, KUSs protected cells from several cell death-inducing insults. For example, when HeLa cells were cultured under low glucose conditions (0.2 g/l of glucose), all cells died within several days (Fig. 2a). However, when KUSs were present in the media, they prevented cell death (Fig. 2a and b). Protective effects were also observed when HeLa cells were treated with tunicamycin (Tm) (Fig. 2c), or when HEK293 cells were subjected to serum-free conditions (Fig. 2d). These protective effects of KUSs were dose-dependent, as exemplified by KUS121 (Fig. 2e). These data clearly implied that inhibition of VCP ATPase activity by KUSs could protect cells from several cell death-inducing insults.

Tunicamycin treatment and glucose starvation are known to cause ER stress and to lead to cell death. C/EBP-homologous protein (CHOP) is a core mediator of ER stress-induced cell death, and is upregulated during ER stress^15^. Indeed, KUSs suppressed the expression of CHOP in tunicamycin-treated HeLa cells (Fig. 2f). KUSs also suppressed the expression of 78 kDa glucose-regulated protein (Grp78), another ER stress marker^16^, in the tunicamycin-treated cells. Next, we examined Akt (serine/threonine-protein kinase) activation by examining its phosphorylation at Ser473, which has been reported to be necessary and sufficient for cell survival^17^. The phosphorylated Akt signal nearly disappeared in cells treated with tunicamycin, but was clearly detected in cells treated with tunicamycin and KUSs (Fig. 2f). These data indicate that KUSs could suppress ER stress and promote cell survival, which was evidenced by Akt being in an activated state.

**Figure 3:**
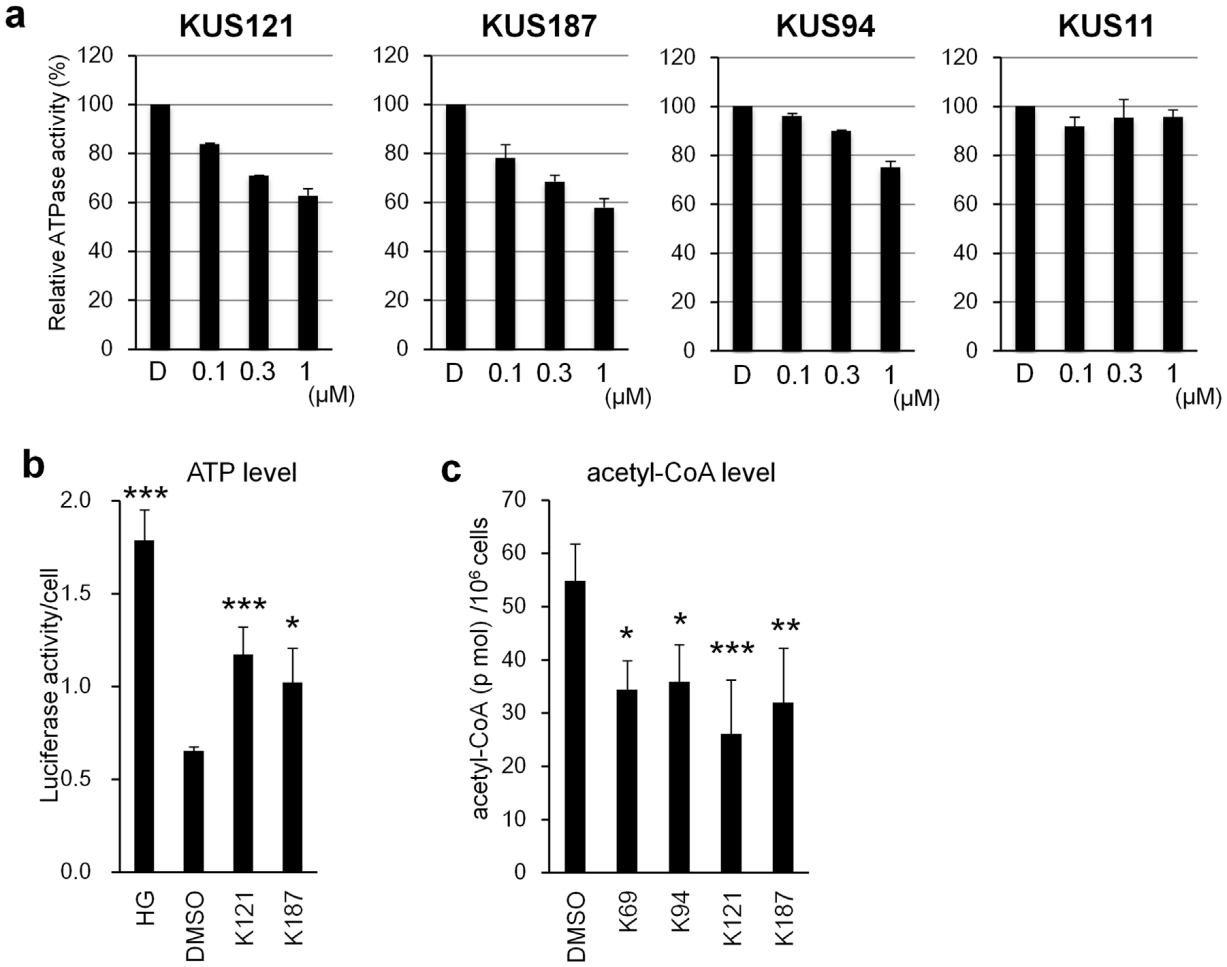
Effects of KUSs on ATPase activities, ATP levels, and acetyl-CoA levels. (a) Inhibition of ATPase activity in clarified whole cell lysates by KUS121, KUS187, and KUS94, but not KUS11. Total ATPase activities in clarified whole cell lysates from differentiated PC12 cells were measured in the absence and presence of KUSs. Relative ATPase activities are shown with values in the absence of KUS (D: DMSO) set at 100%. (b) HeLa cells were cultured in medium with low glucose (0.25 g/l) for 20 hours, with or without KUSs, and ATP levels were measured with luciferase assays. (c) HeLa cells were cultured in medium with low glucose (0.25 g/l) for 20 hours, with or without KUSs, and acetyl-CoA levels were measured. * *P* < 0.05, ** *P* < 0.01, *** *P* < 0.001 vs. DMSO control (Dunnett's test, n = 3).

Because VCP is a major ATPase in cells, we then examined the contribution of VCP to total ATPase activities in clarified whole cell lysates, and found that VCP appeared to contribute to approximately 20%–40% of the total collective ATPase activity, depending on the cell types and culture conditions (an example is provided in Fig. 3a). In neuronally differentiated PC12 cells, for example, 100 nM and 1 μM KUS121, as well as KUS187, significantly lowered the total ATPase activity (approximately 20% and 40% for both concentrations, respectively) (Fig. 3a). Given that the IC_50_ values of KUS121 and KUS187 on recombinant VCP were around 200 ~ 300 nM (Fig. 1a), these data implied that the ATPase activity of VCP contributed to as much as 40% of the total soluble ATPase activities in the cell lysate. This estimation was further supported by the observation that 1 μM KUS94 (IC_50_ is 1 μM) also showed approximately 20% suppression of the soluble ATPase activities (Fig. 3a). Consistent with the inability of KUS11 to inhibit the ATPase of VCP *in vitro* (IC_50_ is more than 100 μM), KUS11 did not show any significant inhibition of total ATPase activity in the clarified lysates (Fig. 3a).

We next examined whether KUSs altered cellular ATP levels. At 20 hours after a change to low glucose medium (0.25 g/l), glucose concentrations in the medium approached zero, and ATP levels in the cells (control cells) significantly decreased (Fig. 3b). In contrast, ATP levels in the cells with low glucose medium plus KUSs remained significantly higher than those in the control cells (Fig. 3b and Supplementary Fig. 2a). In addition, in KUS-treated cells, the ratio of ATP to ADP was higher than in the control cells (Supplementary Fig. 2b). These data indicate that KUSs suppress consumption of ATP in cells under stress conditions. Interestingly, acetyl-CoA levels in the KUS-treated cells were significantly lower than those in the control cells (Fig. 3c and Supplementary Fig. 2c), suggesting the possibility that KUSs may induce a metabolic shift from a glycolytic pathway to mitochondrial pathways to produce ATP. This possibility remains to be clarified. The low levels of acetyl-CoA might also contribute to protect cells from cell death, as reported recently^18^.

### KUSs and exogenous ATP both prevented ER stress in cultured cells

**Figure 4:**
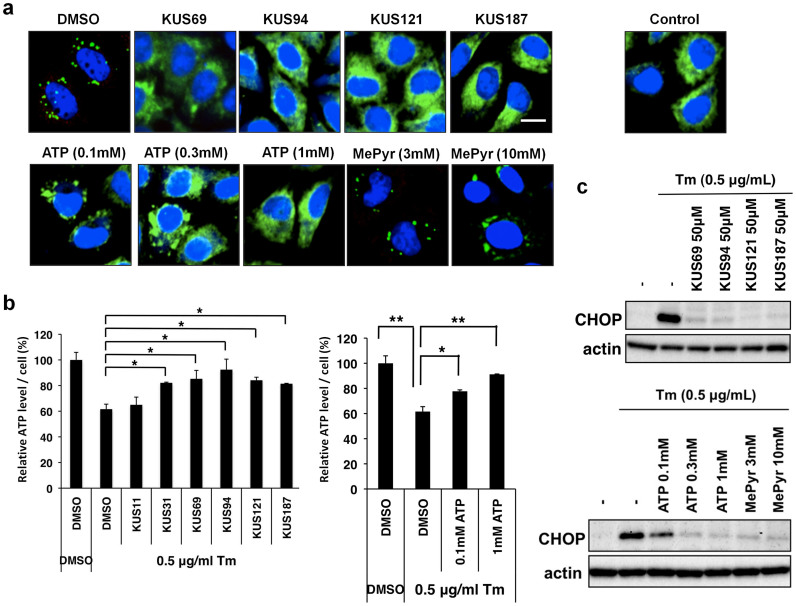
KUSs and ATP each prevented a decrease of ATP levels and ameliorated ER stress in tunicamycin-treated cells. (a) Immunocytochemical analyses of HeLa cells by an anti-laminin γ1 antibody. HeLa cells were treated with 0.5 μg/ml of tunicamycin (Tm) for 5 hours in the presence of KUSs (50 μM), ATP (0.1, 0.3, and 1 mM), methylpyruvate (MePyr) (3 and 10 mM), or vehicle alone (DMSO). Then, cells were fixed and subjected to immunocytochemical analyses. Normally growing HeLa cells were also analyzed (Control). Scale bar, 10 μm. (b) Measurements of the relative amounts of ATP per cell. HeLa cells were treated with tunicamycin (Tm, 0.5 μg/ml) for 24 hours in the presence of KUSs (50 μM) or ATP (0.1 and 1 mM), or vehicle alone (DMSO), and were harvested. Then, ATP amounts from 1.5 × 10^5^ cells were measured^27^. * *P* < 0.05, ** *P* < 0.01. Error bars indicate SD. (c) Western blot analyses on CHOP. HeLa cells were treated with 0.5 μg/ml of tunicamycin for 5 hours in the presence of KUSs (50 μM), ATP (0.1, 0.3, and 1 mM), methylpyruvate (MePyr) (3 and 10 mM), or vehicle alone (-). Then, cells were harvested and subjected to western blot analyses. Actin served as a loading control. Complete scans of the different blots are presented in Supplementary Fig. 9.

It has long been believed that ER stress is induced by the accumulation of misfolded proteins, or protein aggregates, in the ER^19^,^20^,^21^. We recently identified laminin γ1 as an aggregation-prone protein in the ER. We therefore examined laminin γ1 as an indicator of ER-stress in tunicamycin-treated cells. By immunocytochemical analyses, expression of laminin γ1 was observed in a diffuse pattern throughout the ER in normal cells (Control in Fig. 4a). When cells were treated with tunicamycin, laminin γ1 formed clear aggregates (DMSO in Fig. 4a). However, not only 50 μM KUSs (KUS69, 94, 121, and 187) but also 1 mM ATP treatments clearly prevented the aggregation of laminin γ1 (Fig. 4a). Consistent with these results, KUSs and ATP treatments similarly prevented decreases of ATP levels in tunicamycin-treated cells (Fig. 4b). By contrast, KUS11, which could not inhibit VCP ATPase, was unable to prevent the tunicamycin-elicited ATP decrease (Fig. 4b). It is notable that the addition of 0.1 mM ATP or 3 to 10 mM methylpyruvate (weakly membrane-permeable pyruvate, which is converted to ATP in mitochondria) were also ineffective in preventing the aggregation of laminin γ1 (Fig. 4a), but could nevertheless dampen the induction of ER stress, namely CHOP induction (Fig. 4c). These results indicated that the ER is more sensitive to decreases in ATP levels than to the presence of aggregates (see Discussion).

### KUSs mitigated pathologies of rd10, a mouse model of retinitis pigmentosa

As we have long been seeking a new strategy to protect retinal neuronal cells, we were intrigued by the observation that VCP was highly expressed in all types of retinal neuronal cells (Supplementary Fig. 3). Furthermore, in retinitis pigmentosa, an involvement of ER stress has been proposed^2^,^3^. After confirming the neuroprotective efficacy of KUS in retinal organ culture (Supplementary Fig. 4), we then examined whether the protective effects would be observed *in vivo* against the degeneration of photoreceptor cells. For this purpose, rd10 mice were used as a representative mouse model of retinitis pigmentosa^22^. Rd10 mice have a mutation in a gene encoding the rod cyclic guanosine monophosphate (cGMP) phosphodiesterase beta subunit (PDE6B)^22^, which is also mutated in patients with retinitis pigmentosa. The mice have been commonly used to test the efficacies of new treatments, including gene therapy, neuroprotectants, and stem cell derived retinal cells.

**Figure 5:**
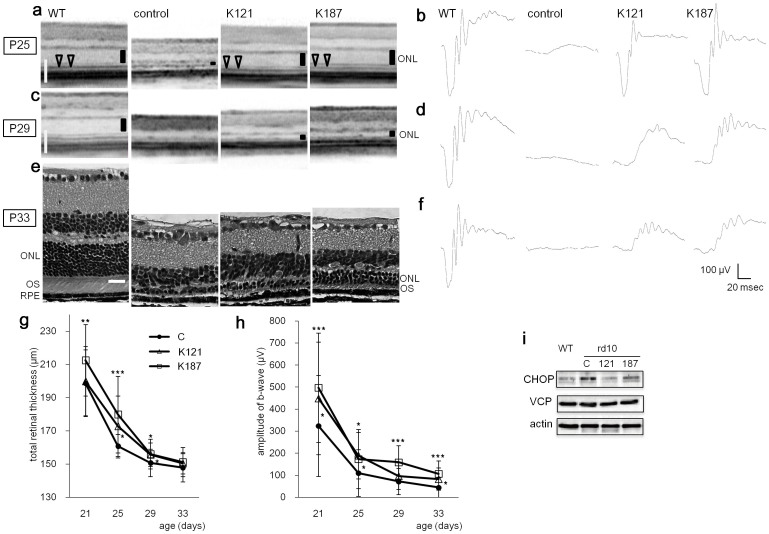
*in vivo* efficacies of KUSs in the rd10 mouse model of retinitis pigmentosa. (a, c) Representative live sectional images (vertical sections) by SD-OCT of retinas in 25- (a) and 29-day-old (c) normal C57BL/6 mouse (WT) and rd10 mice, administered KUS 121 (n = 17), KUS187 (n = 21), or saline (n = 18) as a control. Vertical bars in the images indicate the thickness of outer nuclear layer (ONL). Note that the ONL was barely detectable in saline-treated control rd10 mice. (b, d, f) Electroretinogram of 25- (b), 29- (d) and 33-day-old (f) normal C57BL/6 mouse (WT) and rd10 mice, administered KUSs or saline. (e) HE-stained retinas of 33-day-old normal C57BL/6 mouse (WT) and rd10 mice, administered KUSs or saline. RPE: retinal pigment epithelium. OS: outer segment. Scale bars (shown by white color), 100 μm in (a) and (c); 20 μm in (e). (g, h) Time-dependent changes of total retinal thickness (g) and b-wave amplitude in dark-adapted electroretinogram (h) in rd10 mice administered KUSs or saline. * *P* < 0.05, ** *P* < 0.01, *** *P* < 0.005 vs. saline (Dunnett's test). Error bars indicate SD. (i) Western blot analysis of dissected retinas of 21-day-old rd10 mice administered KUSs or saline. WT: C57BL/6 control mice. Complete scans of the different blots are presented in Supplementary Fig. 10.

**Figure 6:**
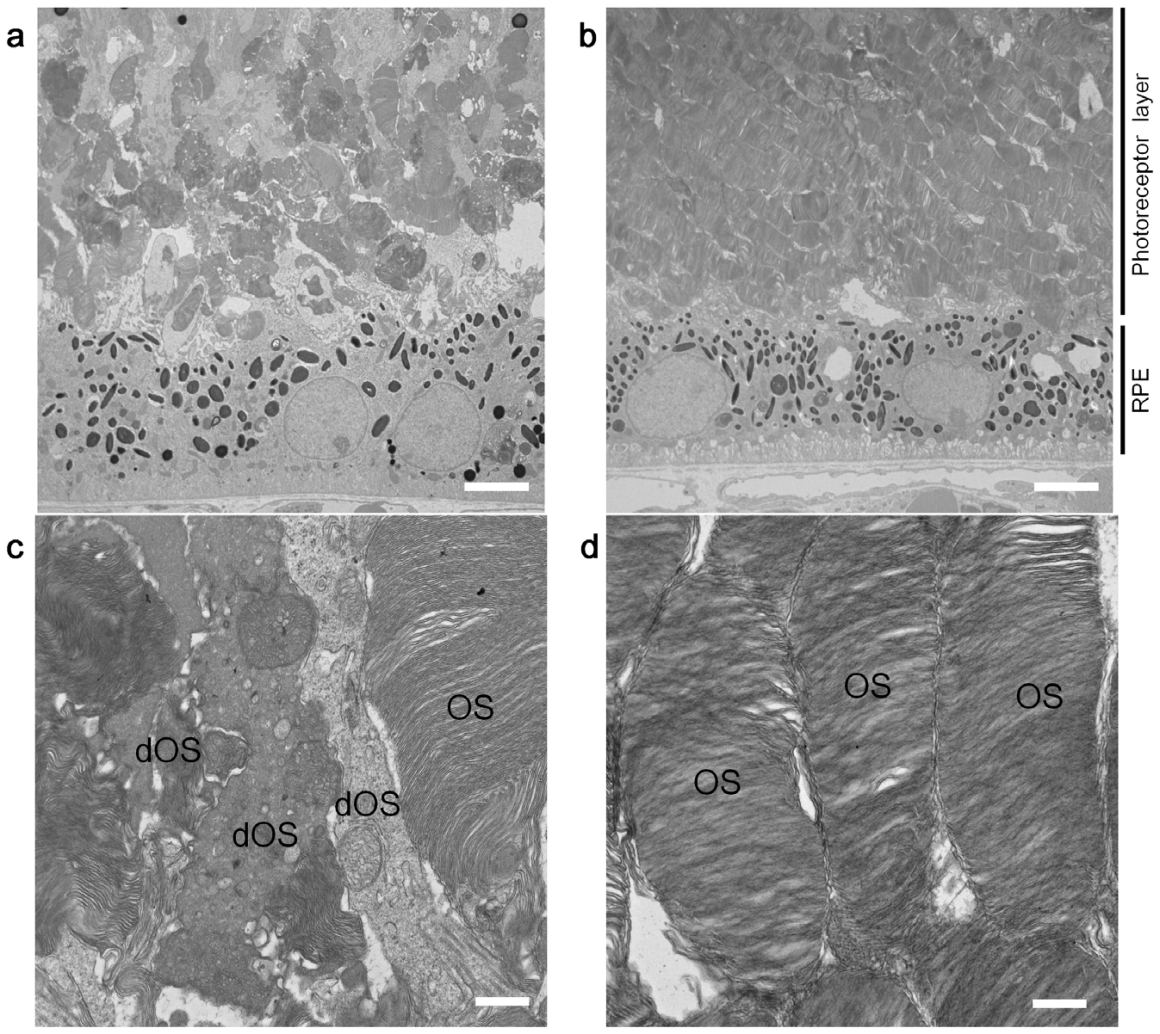
KUSs preserved retinal microstructures in the rd10 mouse model of retinitis pigmentosa. Electron micrographs of 21-day-old rd10 mice, administered saline (a, c) or KUS187 (b, d). RPE, retinal pigment epithelium. Note that morphologies of outer segments of retinas in KUS-treated rd10 mice were well preserved but most of the outer segments in saline-treated control rd10 mice were degenerated. OS in (c) and (d) indicates morphologically intact outer segment, and dOS in (c) morphologically degenerated outer segment. Scale bars: 5 μm in (a) and (b); 500 nm in (c) and (d).

Starting 7 days after birth, KUS121 or KUS187 was administered daily (50 mg/kg) by intraperitoneal injection. Spectral-domain optical coherence tomography (SD-OCT) examination showed that at age 21 days, the retinas of the control rd10 mice had begun to degenerate (Supplementary Fig. 5a). To test the visual function of the mice, dark-adapted electroretinograms were recorded. The amplitude of the a-wave, which represents visual function of photoreceptors, was smaller in the control mice than in the KUS-treated mice (Supplementary Fig. 5b and c). The peak latency of the a-wave, which negatively correlates with visual function of photoreceptors, was delayed in the control mice as compared with the KUS-treated mice (Supplementary Fig. 5d). At age 25 days, the thinning of the outer nuclear layer (ONL) was clearly observed in the control mice (Fig. 5a). The outer nuclear layer and the junction line between the inner segment and outer segment (arrow heads in Fig. 5a), which is generally considered to be positively associated with visual function^23^,^24^, were clearly detected in the KUS-treated but not in control mice. A very small electroretinogram response was observed in control mice, but an almost normal electroretinogram response was observed in most of the KUS-treated mice (Fig. 5b). At age 29 days, the photoreceptor layer was barely detected in SD-OCT images, and electroretinogram records were almost flat in control mice. In the age-matched KUS-treated mice, the outer nuclear layer, although thin, and an electroretinogram response were still observed (Fig. 5c and d). By histological examination, at age 33 days, the outer nuclear layer in the control mice consisted of only 1–2 rows of cells, but there remained 5–6 rows of cells in the outer nuclear layer in the KUS-treated mice (Fig. 5e). In KUS-treated but not control mice, the outer segment of the photoreceptors was observed (Fig. 5e). The electroretinogram was non-recordable in the control mice, but small b-wave and oscillatory potentials were observed in the KUS-treated mice (Fig. 5f). Time-dependent changes in total retinal thickness measured on SD-OCT images (Fig. 5g) and in b-wave amplitudes of dark-adapted electroretinogram (Fig. 5h) showed that KUS treatments had the potential to prevent or delay the disease progression. b-wave amplitudes of dark-adapted electroretinograms in non-treated wild-type mice mostly remained unchanged or slightly increased at the age of 33 days (Supplementary Fig. 6a). Note that KUS administration in adult wild-type mice did not induce any significant change in the amplitude of a- and b-waves (Supplementary Fig. 6b and c). When examined by electron microscopy, the outer segment of the control mouse retina was mostly disarranged at the age of 21 days (Fig. 6a and c), whereas that of KUS-treated mouse retina was regularly arranged (Fig. 6b and d). In rd10 mice, KUS treatments apparently suppressed CHOP expression, as observed in cultured cells (Fig. 5i).

## Discussion

We successfully developed novel ATPase inhibitors for VCP, with a naphthalene-derived structure in common, and we collectively called them KUSs (Kyoto University substances). KUSs showed IC_50_ values from 100 nM to 1 μM for the inhibition of ATPase activities of recombinant VCP *in vitro*. Totally different from known ATPase inhibitors for VCP, e.g. DBeQ^14^, NMS-873^25^, etc., KUSs, e.g. KUS31, KUS69, KUS94, KUS121, KUS187, etc., did not manifest any apparent cellular toxicity, up to 50 μM, on essentially all tested cultured cells; nor did they elicit any aberrant phenotypes that would be expected from the inhibition of cellular VCP functions. These results demonstrated that KUSs could inhibit VCP ATPase activity without inhibiting cellular VCP functions. AAA ATPases might have additional functions that are independent of their ATPase activity. Recently, Noi et al. analyzed the natural movement of recombinant VCP by high-speed atomic force microscopy and demonstrated that ATP-binding mutants of VCP did not display any apparent rotational movement in solution, but wild-type VCP and ATP-hydrolysis mutants of VCP were indistinguishably capable of rotational movements^26^. These data are consistent with the idea that, for at least some VCP functions, ATP binding is essential but ATP hydrolysis is not. This idea is reminiscent of G proteins and actin, whose functions require guanosine triphosphate (GTP) and ATP binding, respectively, but not GTP and ATP hydrolysis, respectively. KUSs likely inhibit the ATPase activity of VCP, but not necessarily VCP functions related to binding of ATP. We thus categorized KUSs as “VCP modulators” rather than “VCP inhibitors”.

Surprisingly, KUSs were able to reduce by approximately 40% the total ATP consumption in whole cell soluble lysates of neuronally differentiated PC12 cells, raising the possibility that VCP accounts for approximately 40% of the ATP consumption among soluble ATPases in the non-dividing PC12 cells. This result led us to speculate that KUSs would significantly reduce ATP consumption in living cells as well. Indeed, in cultured cells, KUSs were shown to maintain ATP levels in starved conditions as well as in conditions with enhanced ATP consumption, such as in tunicamycin-induced ER stress^27^. Consistently, and surprisingly, KUSs and ATP similarly suppressed tunicamycin-induced ER stress and eventually cell death. More surprisingly, KUSs (at 50 μM) and ATP (at 0.3 to 1 mM) similarly prevented the aggregation of laminin γ1 in tunicamycin-treated HeLa cells. It is noteworthy that a low level of ATP (at 0.1 mM) and methylpyruvate (at 3 to 10 mM) were not able to prevent the aggregation of laminin γ1, but were nevertheless able to reduce the induction of CHOP, a well-known ER stress marker, in tunicamycin-treated cells. This observation clearly supports the idea that ER stress is more directly elicited by a decrease of ATP than the presence of aggregated proteins in the ER. Historically, most ER chaperones were originally identified as proteins induced by glucose starvation and thus were named as GRP (glucose-regulated proteins)^28^. Moreover, it has recently been shown that the binding of ER stress sensors, such as PRKR-like endoplasmic reticulum kinase (PERK) and inositol-requiring enzyme-1 (Ire1), to Grp-78 (Bip) is ATP-dependent^29^. Thus, it is likely that a decrease in the ATP level in the ER induces the dissociation of Bip from the ER-stress sensors, leading to their self-oligomerization and subsequent activation.

The evidence that KUSs could prevent the decrease in ATP level in response to several cell death-inducing insults, and eventually cell death, led us to examine the possibility that KUSs would function as cell-protecting compounds in pathological conditions, and that the prevention of early cell death could in turn prevent or delay the deterioration of the affected organs. For this purpose, we chose rd10, a mouse model of retinitis pigmentosa. Currently, it is very difficult to quantitatively measure local ATP levels, and thus we examined whether KUSs could prevent neuronal cell death in the affected retinas.

In rd10 mice, in which the rod cGMP phosphodiesterase beta subunit (PDE6B)^22^ is mutated, KUSs significantly retarded the progress of photoreceptor cell death, and protected the photoreceptor cells morphologically as well as functionally. Reduction of ER stress would be a likely mechanism for KUS-mediated protection of photoreceptors, although our current data are not sufficient to exclude other yet-unknown possibilities. In retinitis pigmentosa, neuroprotective treatment is regarded as an important future therapeutic strategy, and several clinical trials to prolong the viability of the retinal neuronal cells have been ongoing^1^. We are also planning to initiate clinical studies using KUSs in the near future.

Considering all of the data together, we posit that a reduction of ATP levels is a common condition in the affected organs of incurable disorders with early cell death. Because many proteins require ATP, a reduction of ATP levels would contribute to a functional decline in affected cells or organs in early stages of the disease, which might precede cell death. Reducing ATP consumption by way of KUSs and/or enhancing ATP generation by yet-unknown compounds would be a novel strategy to retard these processes and thus to prevent or retard the progression of clinical manifestations. Recently, the involvement of translational suppression via phosphorylation of eIF2α has been proposed in the pathogenesis of Alzheimer^30^ and prion diseases^31^. Indeed, novel PERK inhibitors showed significant efficacies in a mouse prion disease model^32^. Given that PERK is the kinase responsible for the phosphorylation of eIF2α in ER stress, the effect of KUSs in these disease models would be worth evaluating.

In conclusion, we showed that KUSs, new compounds developed as ATPase inhibitors of VCP, have novel functions as “VCP modulators” or “ATP regulators” without apparent inhibition of cellular VCP functions. These new “ATP regulators” have strong neuroprotective effects *in vivo* on retinal photoreceptor cells. The efficacies were apparently correlated with their abilities to suppress ER stress. To the best of our knowledge, KUSs are the first small chemicals that can prevent cell death in an animal model of human retinitis pigmentosa. Given that the major pathology of many other incurable human disorders, e.g. neurodegenerative diseases, ischemic diseases, etc., is also early cell death, KUSs may provide a novel strategy for cell protection in such incurable disorders.

## Methods

### Cell culture

HeLa, HEK293, and PC12 cells were cultured in Dulbecco's modified Eagle's medium (DMEM) with 10% fetal bovine serum. Tunicamycin (0.2–2 μg/ml, Nacalai Tesque, Kyoto, Japan) was added to induce ER stress. Viability of cultured cells was measured by formazan production with an ARVO multilabel counter (Wallac), using WST (water soluble tetrazolium salts)-8 reagent (Cell count reagent SF, Nacalai Tesque, Kyoto, Japan). ATP in cultured cells was measured by luciferase activities with an ARVO multilabel counter, using ATP assay reagent for cells (Toyo B-net, Tokyo, Japan). Acetyl-CoA was measured using a PicoProbe Acetyl CoA assay kit (BioVision, CA, USA) with a Spectra Max multilabel counter (Molecular Devices, CA, USA).

### Measurement of ATPase activities

We measured ATPase activities by modifying the molybdate assay, which was as described previously^8^. In the assay, 1 μg of whole cell soluble lysate of neuronally differentiated PC12 cells, or 500 ng recombinant VCP protein, was incubated in 20 μl of the ATPase assay buffer (20 mM HEPES (pH 7.4), 50 mM KCl, 5 mM MgCl_2_) with 100 μM [γ-^32^P]ATP (18.5 GBq/mmol) (PerkinElmer) at 37°C for 20 min. After incubation, the reaction was quenched by addition of 200 μl of 8% ice-cold trichloroacetic acid solution with 1 mM K_2_HPO_4_, and then 50 μl of solution A (3.75% ammonium molybdate, 0.02 M silicotungstic acid in 3 N H_2_SO_4_) and 300 μl of *n*-butyl acetate were added to the reaction. The samples were mixed well and centrifuged at 12,000 × g for 1 min. Then, 200 μl aliquots from the upper organic phases were taken and their radioactivity was determined with a liquid scintillation counter for β-radiation, which determined the amounts of ^32^P released.

### Antibodies

Polyclonal antibodies against VCP were developed in our laboratory as described previously^12^. Anti-Grp78, anti-Akt and anti-phospho-Akt (Ser 473) antibodies were purchased from Cell Signaling (MA, USA); anti-tubulin, anti-CHOP and anti-laminin γ1 from Santa Cruz Biotechnology (CA, USA); anti-actin from Chemicon (MA, USA); anti-ubiquitin from Millipore (MA, USA), and anti-LC3 from MBL (Nagoya, Japan).

### Animal experiments

Animal experiments were conducted in accordance with the Association Research in Vision and Ophthalmology (ARVO) Statement for the Use of Animals in Ophthalmic and Vision Research. All protocols were approved by the Institutional Review Board of the Kyoto University Graduate School of Medicine (MedKyo11229). Rd10 mice^22^ were obtained from the Jackson Laboratory (Bar Harbor, ME, USA). The environment was maintained at a 14-hour light/10-hour dark cycle. All mice were fed ad libitum. Before image acquisition or electroretinogram examination, the mice were anesthetized by an intraperitoneal injection of pentobarbital (50 mg/kg body weight). Pupils were dilated to approximately 2 mm in diameter using tropicamide and phenylephrine (0.5% each) eye drops.

### SD-OCT image acquisition and measurement of retinal thickness

Spectral-domain optical coherence tomography (SD-OCT) examinations using *Multiline OCT* (Heidelberg Engineering, Heidelberg, Germany) were performed^33^ in rd10 mice at ages of 21, 25, 29, and 33days. Total retinal thickness (from inner limiting membrane to Bruch membrane) in rd10 mice was measured using volume scan images^33^ within a circle 0.366 mm in diameter, the center of which was adjusted to the center of the optic nerve head. The mean value of the upper and lower quadrant was averaged.

### Electroretinogram

Electroretinogram recording was performed to assess the visual function of rd10 mice at ages of 21, 25, 29, and 33 days. Mice were dark-adapted overnight before anesthetization. Electroretinograms were recorded using a gold loop corneal electrode with a light-emitting diode (Mayo Corp., Inazawa, Japan). A reference electrode was placed in the mouth and a ground electrode was inserted to the anus. Stimuli were produced with a light emitting diode stimulator (Mayo Corp.). The electroretinogram response signals were amplified, digitized at 10 kHz with a band-pass filter of 0.3 to 500 Hz, and analyzed (PowerLab 2/25; AD instruments, New South Wales, Australia). The a- and b-wave amplitudes and a-wave latency of the mixed cone and rod response (ISCEV (International Society for Clinical Electrophysiology of Vision) standard; scotopic 3.0)^34^ were analyzed.

### Histological analyses

The eyes were fixed in 4% paraformaldehyde for 24 hours at 4°C and embedded in paraffin. Serial 6-μm paraffin-embedded sections that passed through the center of the optic nerve head were selected. The selected retinal sections were stained with hematoxylin-eosin (HE) and photographed about 400 μm apart from the center of the optic nerve head under an optical microscope (Axioplan 2; Carl Zeiss Jena GmbH, Jena, Germany). For electron microscopic examination, eyes were fixed overnight in a mixture of 10% neutral buffered formalin and 2.5% glutaraldehyde for 2.5 hours and subsequently fixed in 1% osmium tetroxide for 90 min. The retina was dehydrated through a graded series of ethanol (50–100%), cleared in propylene oxide, and embedded in epoxy resin. Ultrathin sections were cut by using an ultramicrotome and stained with uranyl acetate and lead citrate. The stained sections were observed by transmission electron microscopy (H-7650, Hitachi Co., Tokyo, Japan).

### Statistical analysis

Variables among cells or mice treated with or without KUSs were compared with Dunnett's test or Student's *t*-test. Statistical analyses were performed using PASW Statistics version 17.0 (SPSS Inc., Chicago, IL). The level of statistical significance was set at *P* < 0.05.

## Author Contributions

H.O.I. designed and conducted the majority of the animal experiments, and prepared the manuscript. N.S., M.K., S.H. and A.I. conducted experiments with cultured cells. N.N. conducted mouse experiments. Y.M. conducted some of the mouse experiments and electron microscopic examination. Y.T. made histological sections. T.F. and T.S. developed KUSs. N.Y. helped with experimental design. A.K. conceived the project and helped with writing the manuscript. All authors discussed the results and commented on the manuscript.

## Supplementary Material

Supplementary Information Supplementary Figures
