# Supplementary material for: Novel VCP modulators mitigate major pathologies of rd10, a mouse model of retinitis pigmentosa
Source: Sci Rep. 2014 Aug 6;4:5970. doi: 10.1038/srep05970 (PMC4122966; doi:10.1038/srep05970)
Supplement: Supplementary Information — Supplementary Figures [file srep05970-s1.pdf]

Supplementary information

Novel VCP modulators mitigate major pathologies of rd10, a mouse model of retinitis pigmentosa

Hanako Ohashi Ikeda, Norio Sasaoka, Masaaki Koike, Noriko Nakano, Yuki Muraoka, Yoshinobu Toda, Tomohiro Fuchigami, Toshiyuki Shudo, Ayana Iwata, Seiji Hori, Nagahisa Yoshimura & Akira Kakizuka

Supplementary Fig.1

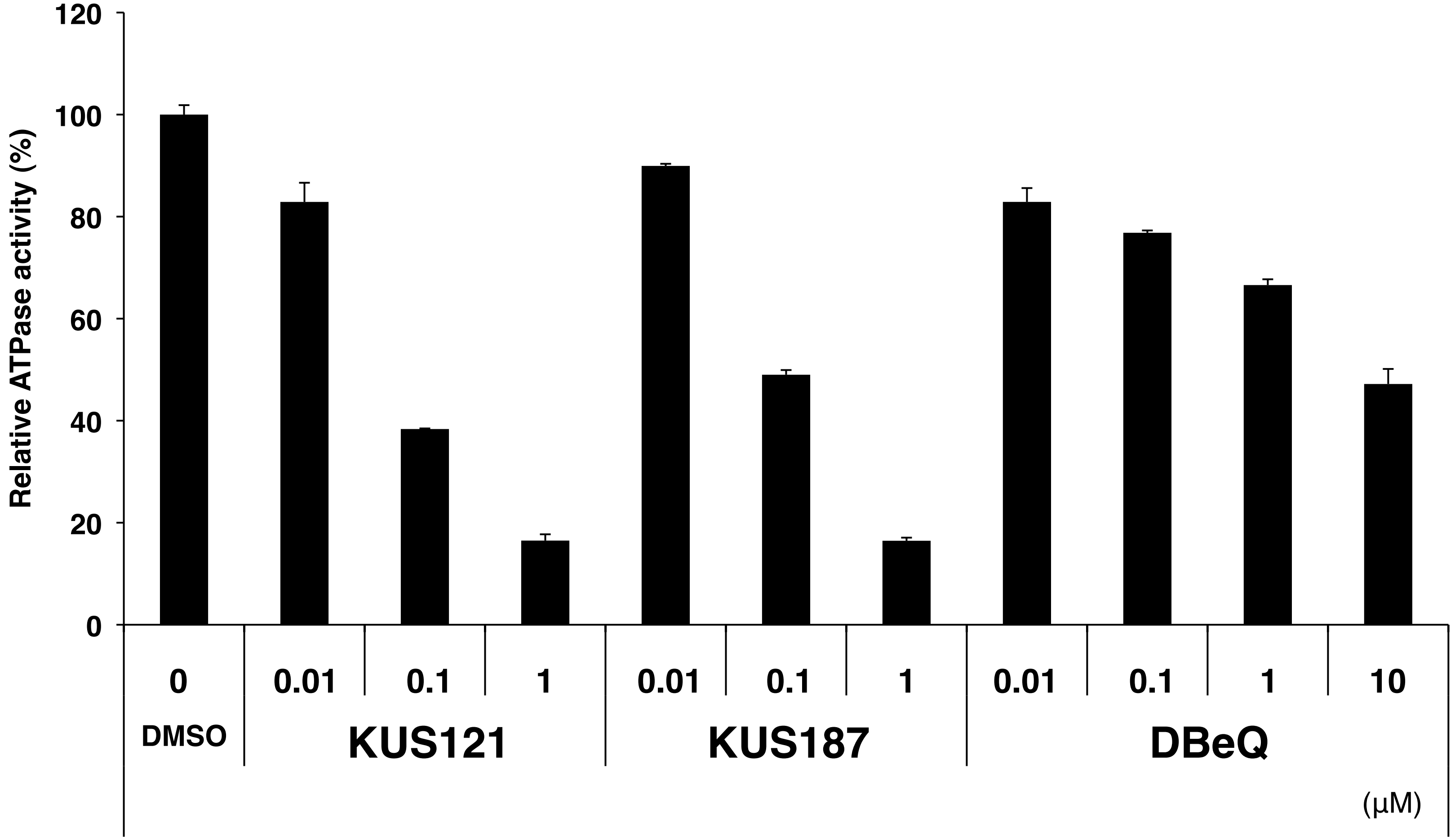

Supplementary Fig.2

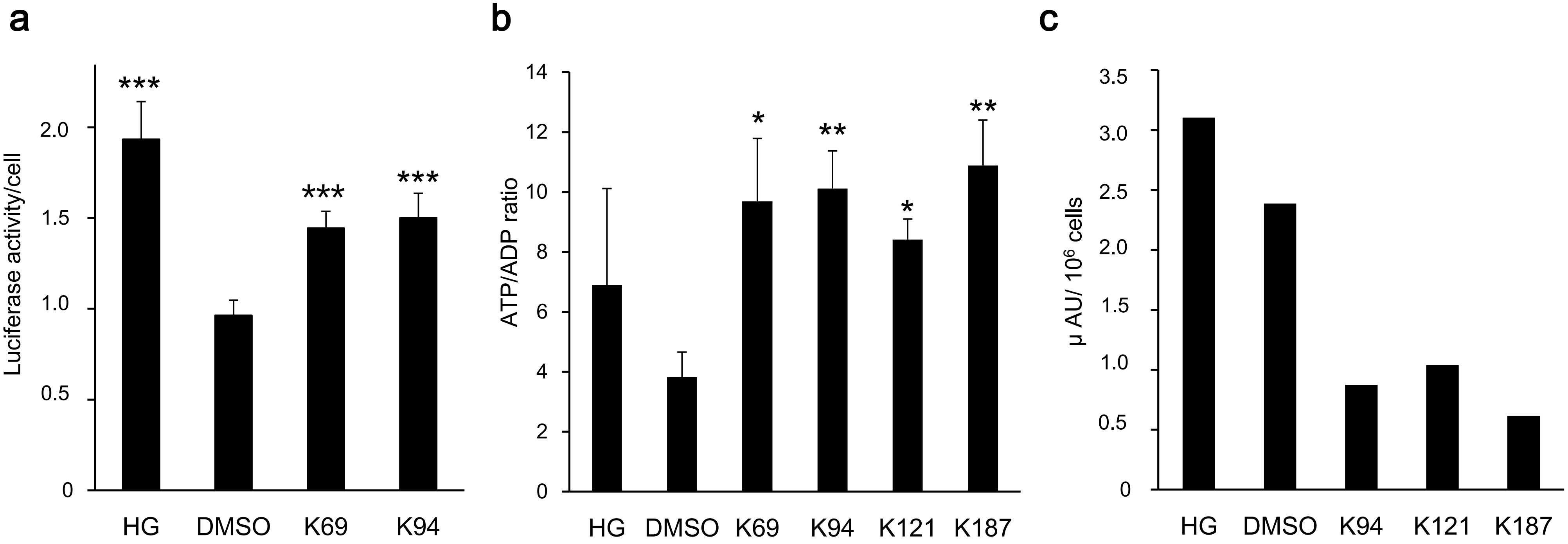

Supplementary Fig.3

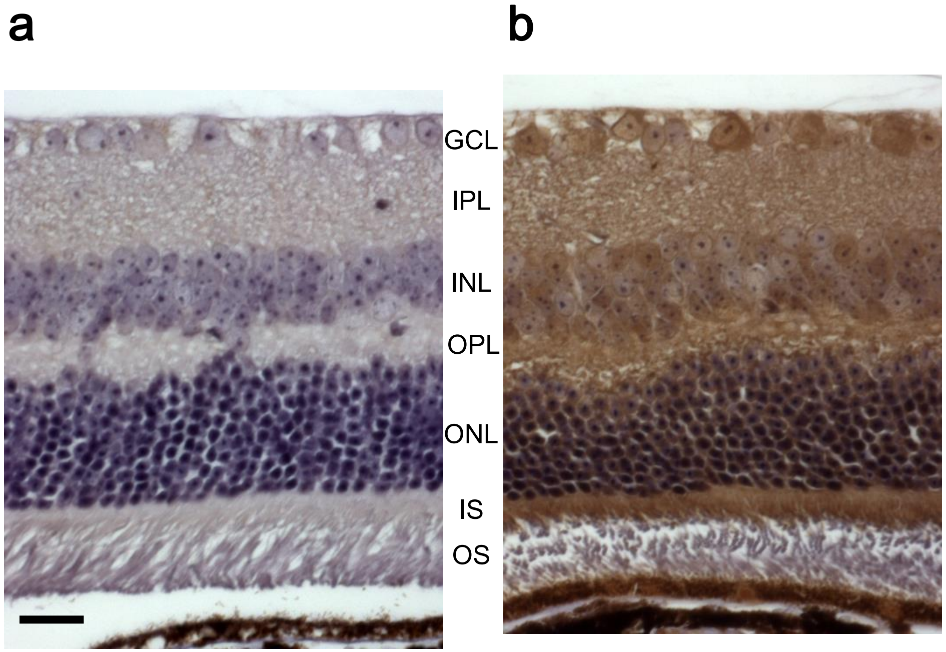

Supplementary Fig.4

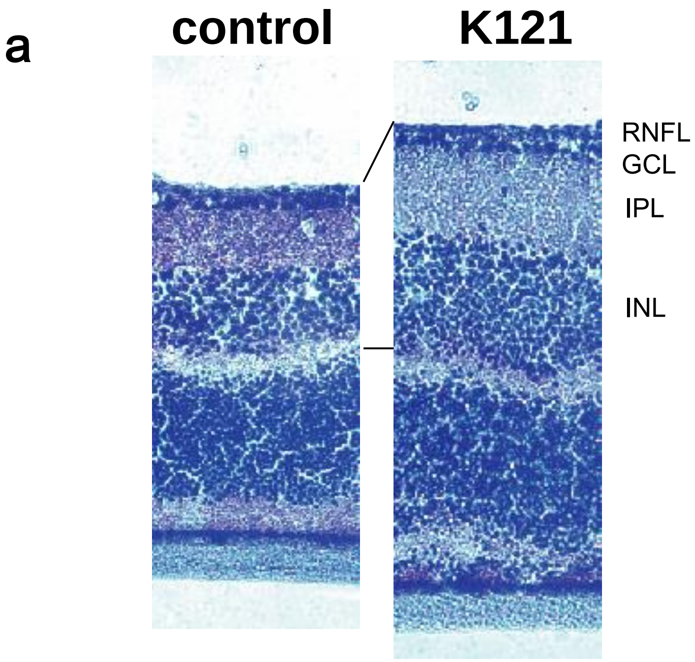

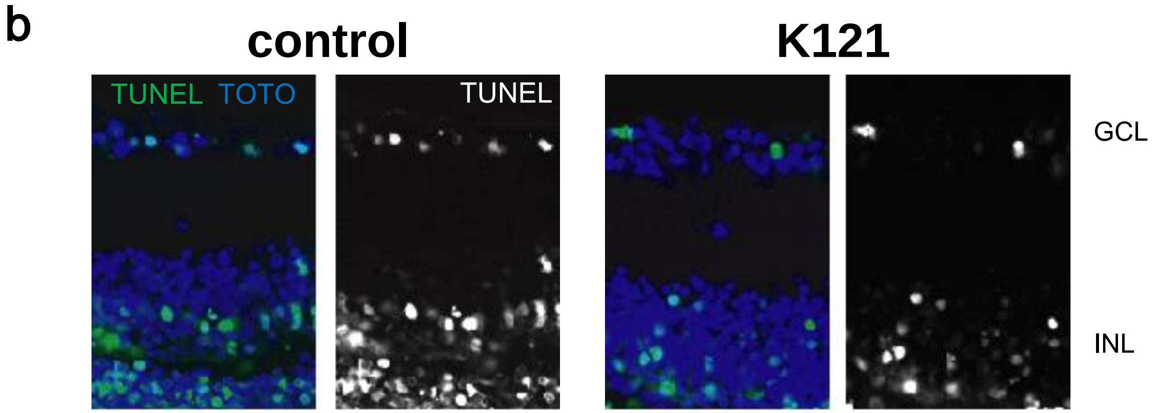

Supplementary Fig.5

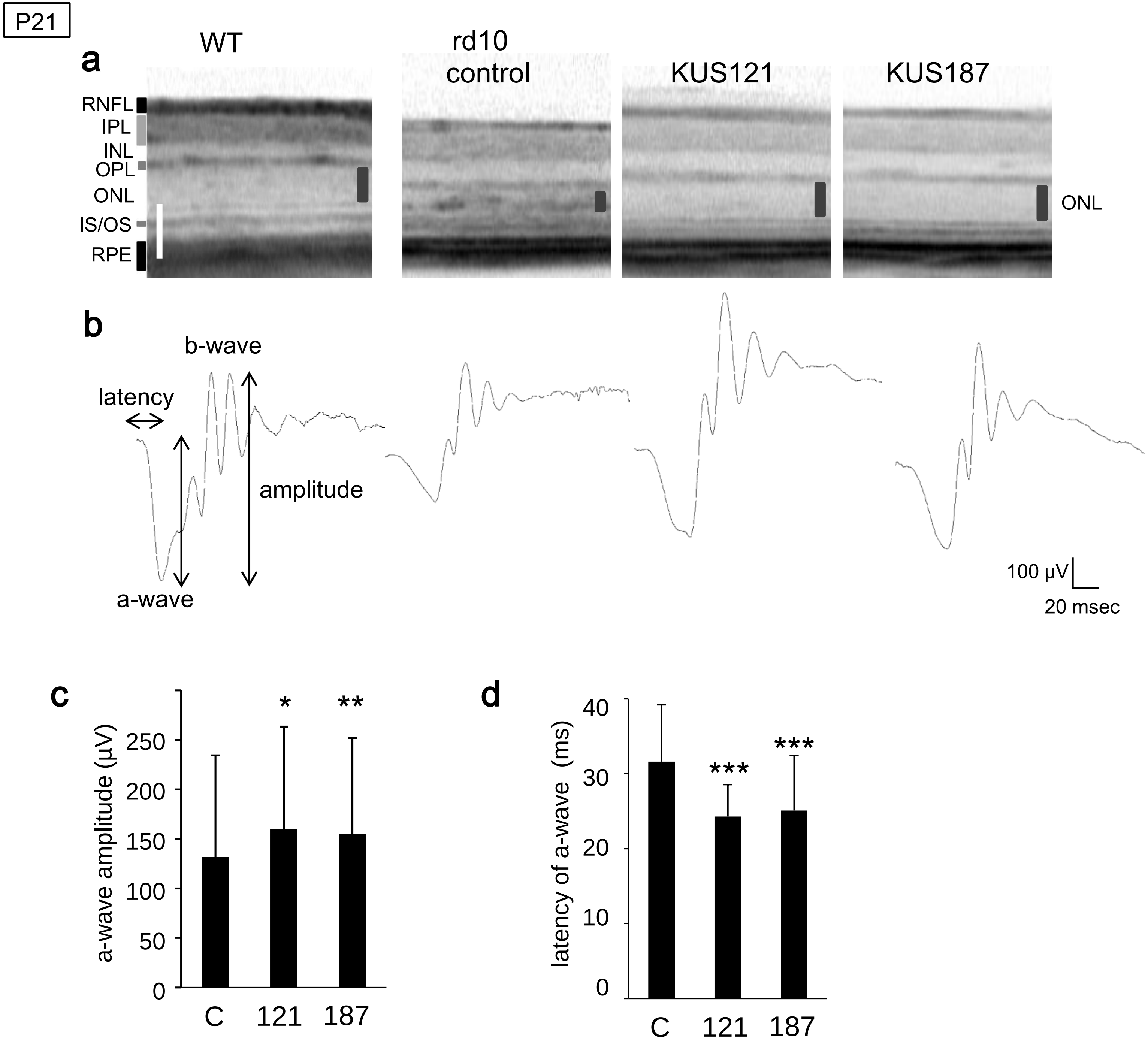

Supplementary Fig.6

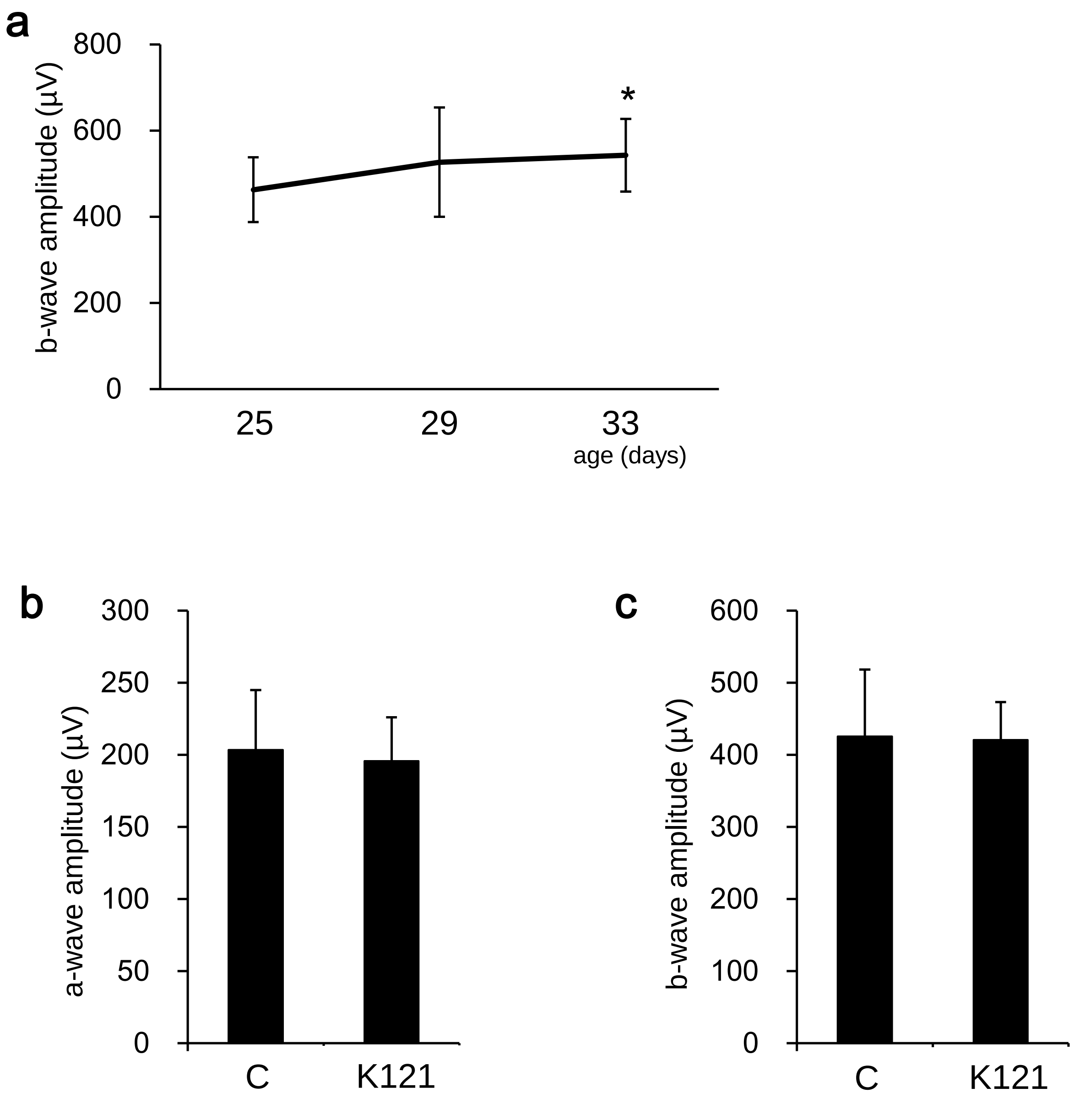

Supplementary Fig.7

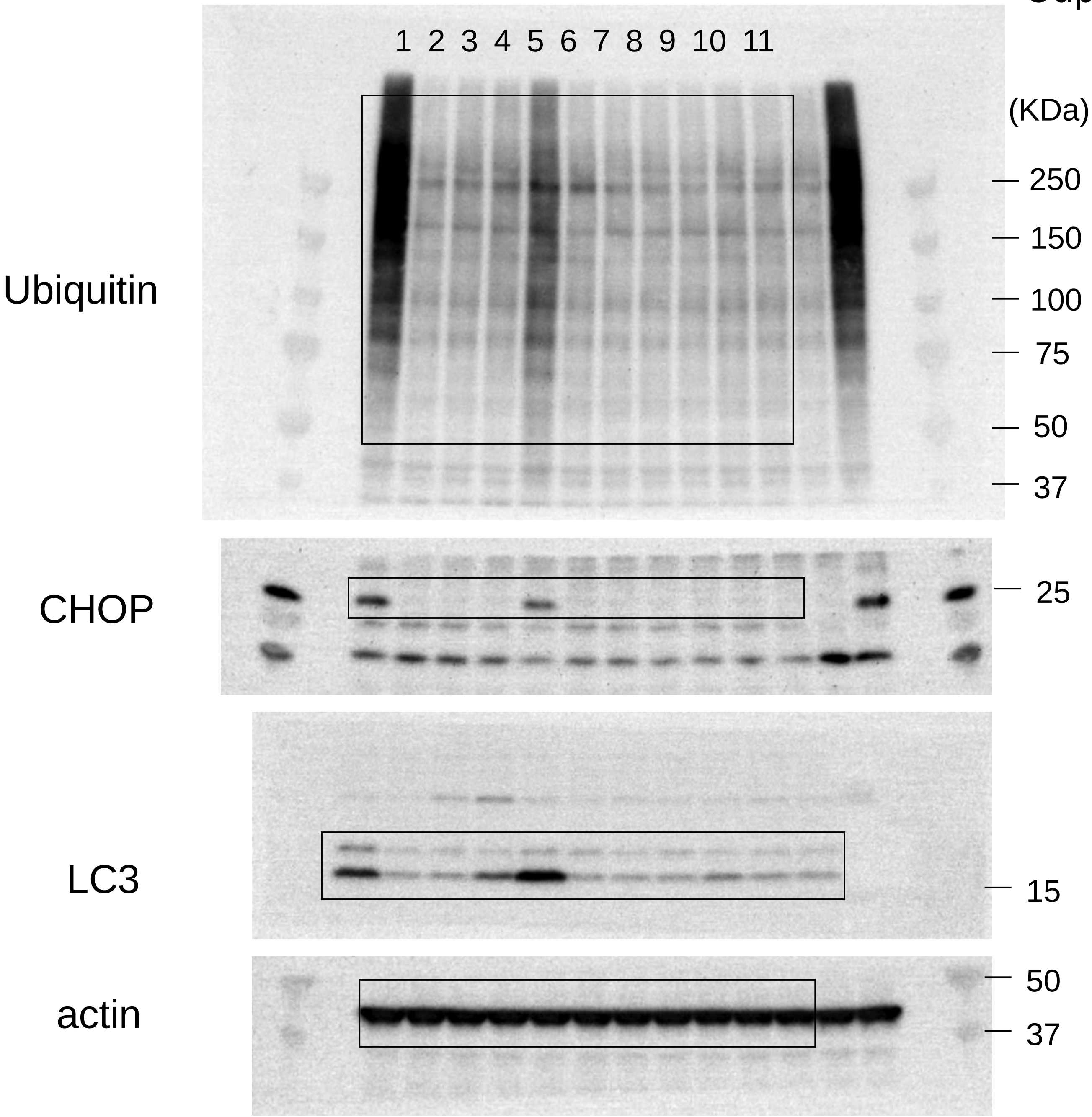

Fig.1c

Supplementary Fig.8

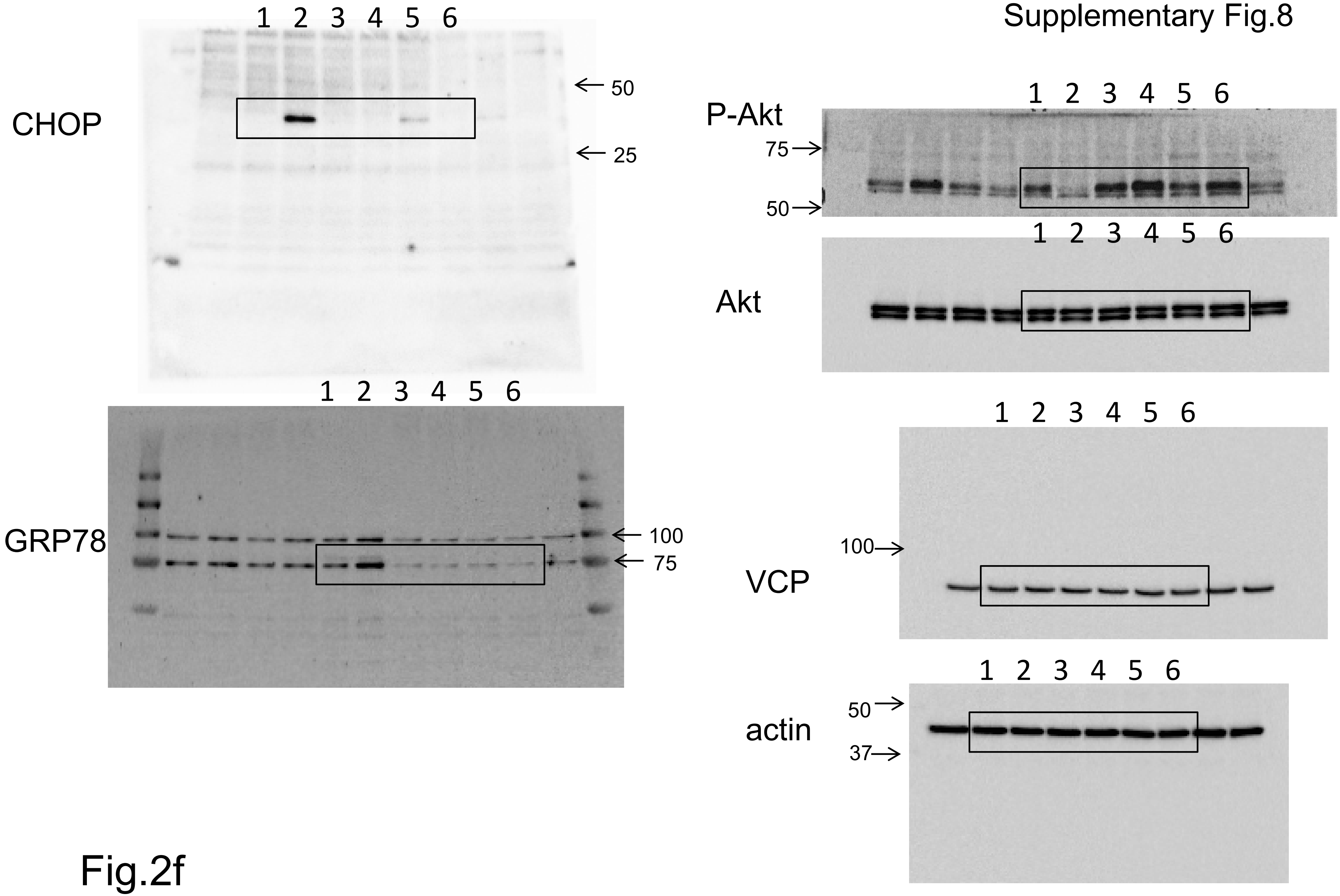

Fig.2f

Supplementary Fig.9

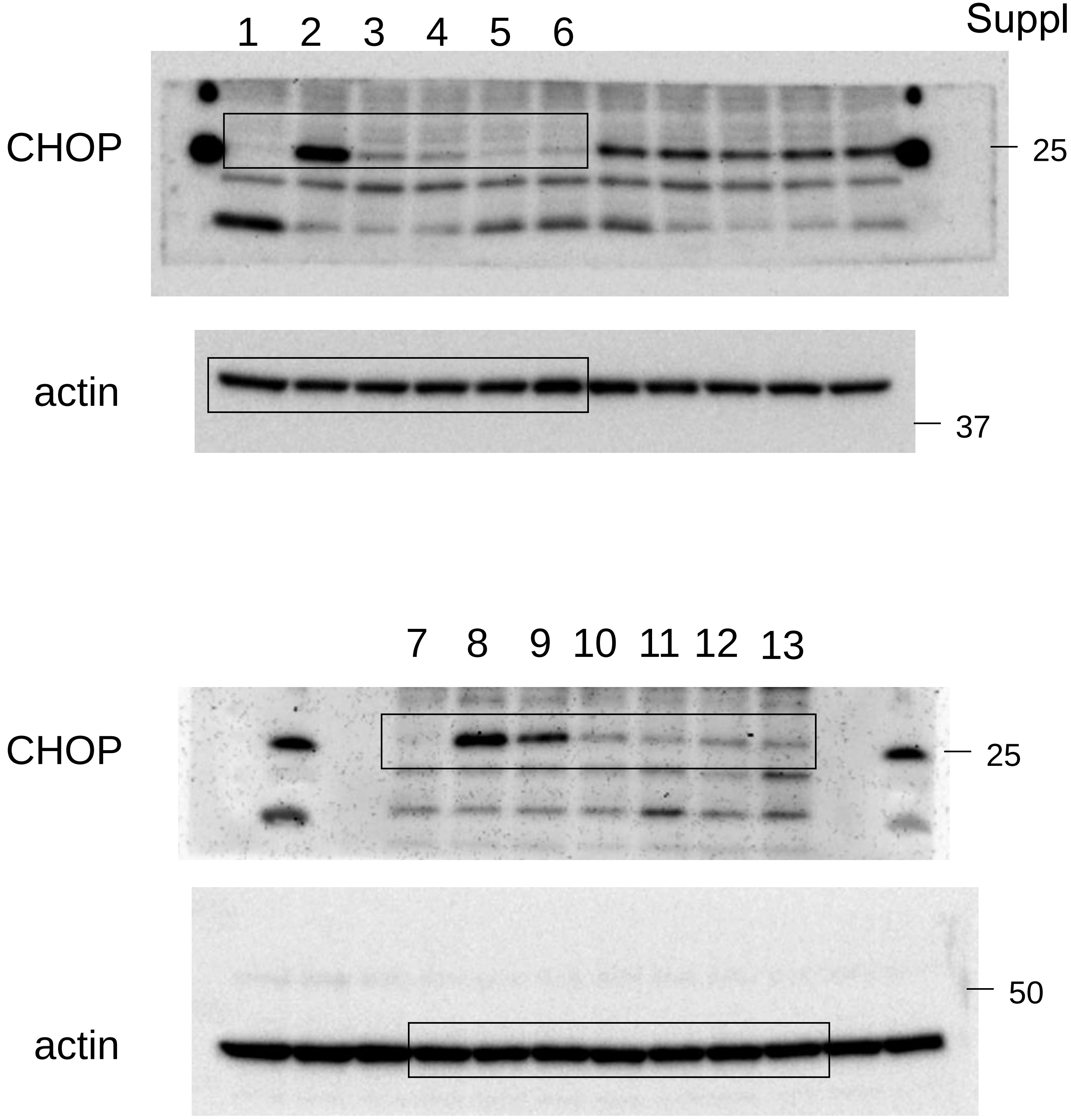

Fig.4c

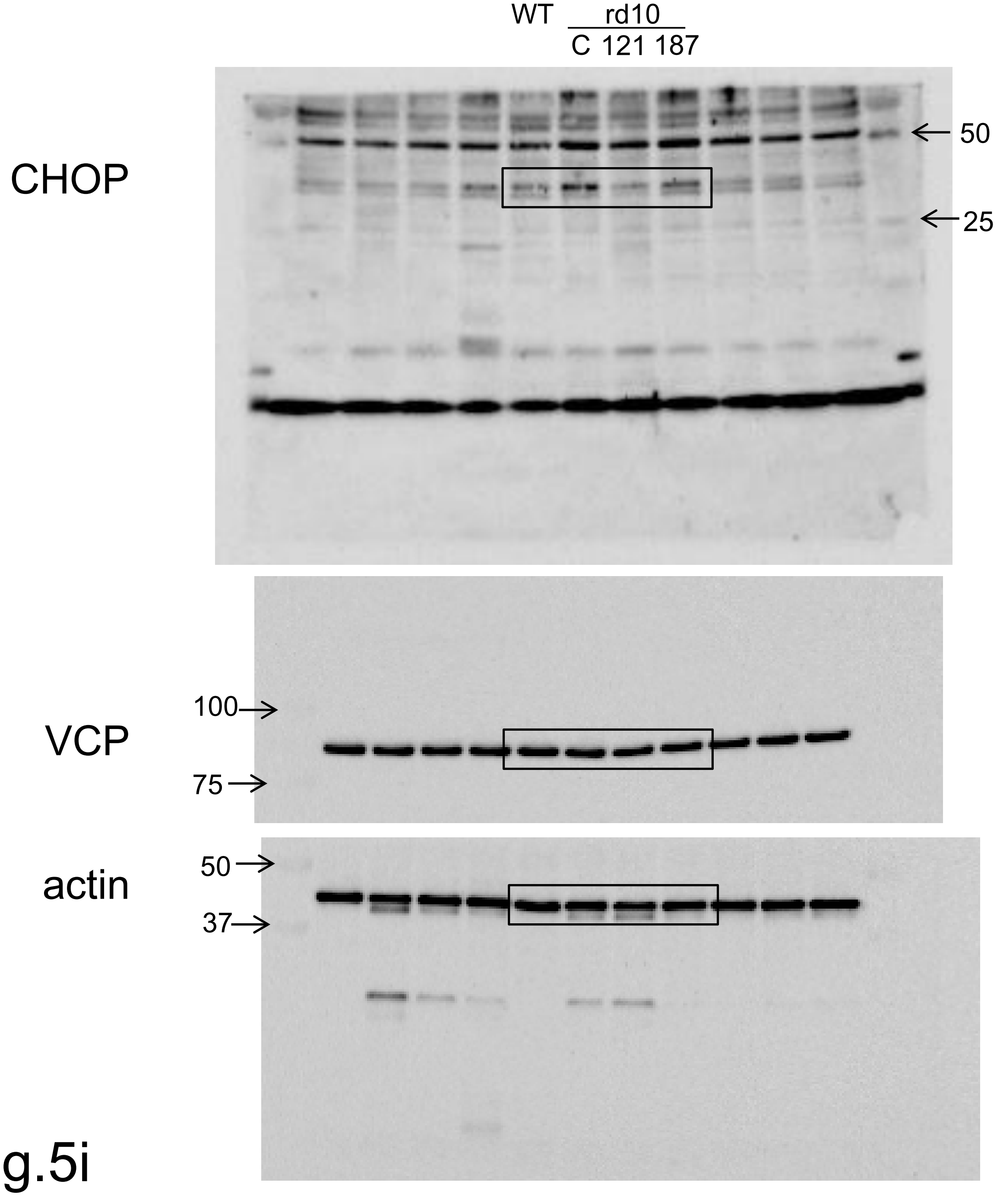

Fig.5i

Supplementary Figure Legends

Supplementary Figure 1 Inhibitory effects of KUS121, KUS187, and DBeQ on the ATPase activity of recombinant VCP purified from E. coli.

Supplementary Figure 2 (**a**) HeLa cells were cultured in medium with low glucose (0.25 g/l) for 20 hours, with or without KUSs, and ATP levels were measured with luciferase assays. *** $P < 0.005$ vs. DMSO control (Dunnett's test, n=3). HG: medium with 4.5 g/l glucose. Error bars indicate SD. (**b**) HeLa cells were cultured in medium with low glucose (0.2 g/l) for 20 hours, with DMSO or KUSs, and ATP and ADP levels were measured by HPLC. The respective ratios of ATP/ADP are shown. * $P < 0.05$, ** $P < 0.01$ vs. DMSO control (Dunnett's test, n=3). Error bars indicate SD. (**c**) HeLa cells were cultured in medium without glucose for 6 hours, with (50 μM each KUS, as indicated) or without KUSs, and acetyl-CoA levels were measured by HPLC.

Supplementary Figure 3 Immunohistochemical analyses of retinal neuronal cells. Retinal sections of 5-month-old C57BL/6 mice were stained with (**b**) or without (**a**) an anti-VCP antibody[12], developed with diaminobenzidine and counter-stained with hematoxylin (**a, b**). Note that all retinal neuronal cells highly expressed VCP. GCL: ganglion cell layer, IPL: inner plexiform layer, INL: inner nuclear layer, OPL: outer plexiform layer, ONL: outer nuclear layer, IS: inner segment, OS: outer segment.

Supplementary Figure 4 KUS121 protects retinal neuronal cells from NMDA insult. Retinas from adult C57BL/6 mice were cultured on porous membrane with 200 μM of NMDA with or without KUS121 (50 μM) for 4 days. (**a**) HE-stained retinal sections. In the retina cultured with KUS121, the inner nuclear layer (INL), inner plexiform layer (IPL), ganglion cell layer (GCL), and retinal nerve fiber layer (RNFL) were thicker than that of the retina without KUS121. (**b**) TUNEL staining of the retinal sections. More TUNEL-positive apoptotic cells (green) were observed in the retina without KUS than with KUS121.

Supplementary Figure 5 (**a**) Cross sectional (vertical) images obtained by spectral-domain optical coherence tomography (SD-OCT) in 21-day-old C57BL/6 wild-type (WT) and rd10 mice with or without KUSs. Vertical bars in the images indicate the thickness of outer nuclear layer. RNFL: retinal nerve fiber layer, IPL: inner plexiform layer, INL: inner nuclear layer, OPL: outer plexiform layer, ONL: outer nuclear layer, IS: inner segment, OS: outer segment, IS/OS: junction line between IS and OS. Scale bar (shown by white color), 100 μm. (**b-d**) Dark-adapted electroretinogram was performed on 21-day-old C57BL/6 wild-type and rd10 mice. (**b**) Representative electroretinogram records are shown. Double-headed arrows indicate amplitudes of a- and b-wave, and latency of a-wave. (**c**) The mean amplitude of the a-wave (measured at 25 msec), which is derived from photoreceptors, in KUS-treated mice was larger than that in control rd10 mice. * $P = 0.045$ and 0.006 in KUS121 and KUS187 vs. control, respectively (Dunnett's test). Error bars indicate SD. (**d**) Latency of a-wave in electroretinogram. In KUS-treated mice, mean latencies were delayed, compared with those in the control mice. *** $P < 0.001$, vs. control (Dunnett's test). Error bars indicate SD.

Supplementary Figure 6 (**a**) Time-dependent changes of b-wave amplitude in dark-adapted electroretinograms in non-treated C57BL/6 wild-type mice. b-wave amplitudes remained unchanged or slightly increased at the ages of 25 to 33 days. * $P = 0.004$. (**b, c**) a-wave (**b**) and b-wave (**c**) amplitude of adult C57BL/6 wild-type mice administered saline or KUS121 for 7 days. There were no significant differences in the amplitudes of a-and b-waves between mice administered saline or KUS121 (a-wave amplitude: 203.5 ± 41.6 vs.195.8 ± 30.2, $P = 0.56$, *t*-test; b-wave amplitude: 425.8 ± 92.9 vs. 420.8 ± 52.6, $P = 0.86$, *t*-test). Error bars indicate SD.

Supplementary Figure 7 Complete scans of the different blots presented in Figure 1c. Lane 1, 1μM MG132; lane 2, control; lane 3, 5μM DBeQ; lane 4, 10μM DBeQ; lane 5, 15μM DBeQ; lane 6: KUS11; lane 7, KUS31; lane 8, KUS69; lane 9, KUS 94; lane 10, KUS121; lane 11, KUS187.

Supplementary Figure 8 Complete scans of the different blots presented in Figure 2f. Lane 1, Tm(-); lane 2, control; lane 3, KUS69; lane 4, KUS94; lane 5, KUS121; lane 6, KUS187.

Supplementary Figure 9 Complete scans of the different blots presented in Figure 4c. Lane 1, Tm(-); lane 2, control; lane 3, KUS69; lane 4, KUS94; lane 5, KUS121; lane 6, KUS187; lane 7, Tm(-); lane 8, control; lane 9, 0.1mM ATP; lane 10, 0.3mM ATP; lane 11, 1mM ATP; lane 12, 3mM methylpyruvate; lane 13, 10mM methylpyruvate.

Supplementary Figure 10 Complete scans of the different blots presented in Figure 5i.
